# Comparison of Genetic Merit for Weight and Meat Traits between the Polled and Horned Cattle in Multiple Beef Breeds

**DOI:** 10.3390/ani11030870

**Published:** 2021-03-18

**Authors:** Imtiaz A. S. Randhawa, Michael R. McGowan, Laercio R. Porto-Neto, Ben J. Hayes, Russell E. Lyons

**Affiliations:** 1 School of Veterinary Science, University of Queensland, Gatton, QLD 4343, Australia; m.mcgowan@uq.edu.au (M.R.M.); russell@agri-geneticsconsulting.com.au (R.E.L.); 2 CSIRO Agriculture and Food, St Lucia, QLD 4067, Australia; laercio.portoneto@csiro.au; 3 Centre for Animal Science, Queensland Alliance for Agriculture and Food Innovation, University of Queensland, St Lucia, QLD 4072, Australia; b.hayes@uq.edu.au; 4 Agri-Genetics Consulting, Brisbane, QLD 4074, Australia

**Keywords:** genetic correlations, production traits, poll breeding, genetic potential, beef cattle

## Abstract

**Simple Summary:**

Beef production has expanded worldwide through cattle adaptation to diverse environmental and husbandry conditions. The beef industry faces societal challenges from animal welfare perspectives, including dehorning and disbudding, which are common farm practices to limit animal and handler injuries by the horned cattle. Most cattle breeds were originally horned, and reluctance to poll breeding existed because of perceived negative correlations between polledness and production. In Australia, population trends indicate a recent rise to above 50% of poll types in six breeds (Charolais, Hereford, Limousin, Simmental, Shorthorn, and Droughtmaster), and two breeds with lower but increasing rates of polledness (Brahman and Santa Gertrudis). Overall, recently estimated breeding values of 12 investigated production traits have not shown consistently negative trends within and across breeds. Thus, polledness should not be considered as detrimental. Cautious breeding plans are warranted to avoid inbreeding depression in breeds with lower poll frequency. These findings should support the augmented poll breeding across the beef industry.

**Abstract:**

Breeding for polled animals is deemed the most practical solution to eradicate horns naturally and circumvent management costs and risks on health and welfare. However, there has been a historical reluctance by some farmers to select polled animals due to perceived lower productivity of their calves. This study has compared estimated breeding values (EBVs) between horned and polled animals (N = 2,466,785) for 12 production and carcass traits to assess historical (before 2000) and recent (2000–2018) genetic implications of poll breeding. Older generations of the polled animals in most breeds had significantly lower (Bonferroni-corrected *p =* 0.05) genetic merits for live (birth to maturity) and carcass weights, milk, meat quality, and fat content traits. Substantial gains of genetic potential were achieved during 2000 to 2018 in each breed, such that polled animals have significantly improved for the majority of traits studied. Generally, polled cohorts showed advantageous EBVs for live and carcass weights irrespective of the lower birth weights in some breeds. While Polled Brahman showed inferior production parameters, the poll genetics’ effect size (*d*) and correlation (*r*) were very small on recent birth weight (*d* = −0.30, *r* = −0.08), 200 days (−0.19, −0.05), 400 days (−0.06, −0.02), 600 days (−0.05, −0.01), mature cow live weight (−0.08, −0.02), and carcass weight (−0.19, −0.05). In conclusion, although there is some evidence that historical selection for polled breeding animals may have reduced productivity, there is strong evidence that more recent selection for polled genotypes in the breeds studied has not resulted in any adverse effects on genetic merit.

## 1. Introduction

Livestock production and associated industries have been expanding globally due to increasing demands for high-quality protein by the rapidly growing human population. The beef sector has been highly dynamic by attaining animal population growth and farm production efficiency in both developing and developed countries [1] through animal breeding and improved feeding and health systems. However, the increasing drive for improved environmental sustainability and animal welfare outcomes have posed new challenges [2,3].

Polledness—the absence of horns—is an example of a recently in-demand trait in cattle for improved animal welfare and economics. Once, horned animals were perceived as genetically superior and were extensively used in breeding programs for dairy and beef production. However, the cattle industry also incurs significant economic costs associated with horns, including extra housing requirements and bruising in feedlots, during transport, and in abattoirs, and fatal dangers to animal handlers [4,5]. Increasing pressure to mitigate economic and safety concerns has promoted different horn management practices to physically dehorn cattle [6]. Nevertheless, dehorning and disbudding (at an early age) methods are costly due to labor costs and calf loss from infection or excess bleeding [7]. Further, the practice is painful and has raised animal welfare concerns [8,9]. Therefore, breeding for naturally polled cattle has been proposed as a plausible solution [10].

In some traditionally horned breeds, poll breeding initially faced resistance for fear of losing earlier genetic gains in production traits achieved by selecting horned animals during the commercial establishment of the specialized breeds [11]. This resulted in a persisting perception among some cattle farmers that horned animals outperform polled animals and a presumption that polledness was genetically antagonistic with other economically important production traits. Genetic effects due to inbreeding have been noticed in dairy cattle because of a few polled elite bulls used through artificial insemination [12,13]. On the other hand, perceptions about the undesirable effects of polledness on beef production have been largely annulled by comparing different traits of growth, carcass yield, and meat quality in a few beef breeds and cross-bred herds [5,14,15,16].

Modern beef production systems have included many new traits and efficient selection tools to assess the management strategies, production performance, and breeding programs across many breeds [17,18]. Therefore, quantifying the implications of poll breeding in a diverse set of beef breeds is possible by using the animals’ genetic merit corresponding to the on-farm recorded traits. For instance, evaluation of genetic effects of a trait is more practical by substituting estimated breeding value (EBV) for a phenotypic value. EBV is a genetic evaluation tool between animals for a particular trait by accounting for heritability and fixed effects [19]. EBVs for a quantitative trait capture the aggregate additive genetic value by using phenotype of an animal together with phenotypes of its relatives [20]. EBVs denote how an animal’s genetics is different than the historic genetic base or breed averages. The accuracy of EBV predictions increases as more information becomes available about the performance of an animal and its progeny [21]. BREEDPLAN (https://breedplan.une.edu.au) is an advanced genetic evaluation system implemented for a national beef recording scheme in Australia to compute EBVs of various economically important traits [19]. It has been utilized by many breeds over decades.

This study was undertaken to compare historic (1950s to 1999) and recent (2000 to 2018) changes in genetic gains for 12 beef production traits (weights at birth, 200 days, 400 days, 600 days, mature cow and carcass, milk, and retail beef yield) and meat quality (eye muscle area, intramuscular, rib, and rump fat). The genetic merit of the horned and polled animals was compared within eight major breeds of Australian beef cattle including *Bos taurus* (Charolais, Hereford, Limousin, Shorthorn, and Simmental), *Bos indicus* (Brahman), and their composites (Droughtmaster and Santa Gertrudis).

## 2. Materials and Methods

### 2.1. Genetic Evaluations of Beef Production and Quality Traits

Genetic merit of beef cattle in Australia is evaluated using Best Linear Unbiased Prediction (BLUP) technology. The BLUP model performs a multi-trait genetic evaluation on a breed-by-breed basis as explained in Reference [19]. Briefly, phenotypic records are pre-corrected for fixed effects like age of dam and age of animals using breed by sex (and calving season)-specific factors. Then, the BLUP model incorporates: fixed effect of contemporary group, random additive genetic effect for animal, random maternal genetic effect for dam, random permanent maternal environment effect for dam, random sire by herd interaction effect, and heterogeneity adjustment based on interaction between herd, year, and sex. In addition, a standard pedigree-based relationship matrix (and genomic relationship matrix for limited breeds) is used to estimate breeding values (EBVs) [22]. Trait-wise EBVs are estimates of an animal’s true breeding values. EBVs are expressed as genetic differences (in trait units) between the animal and a historic group of animals (i.e., genetic base) of the same breed. The genetic base of each breed has been set to a historical benchmark (mid 1990’s), and within breed averages of various EBVs are expected to change over time as a result of genetic progress. BREEDPLAN EBVs are computed for most target traits for animals aged at least two years. Thus, EBVs of animals born up to 2018 were available in 2020. For this study, EBVs of 12 traits representing beef production and quality parameters were obtained. The traits include weights (kg) at birth, 200 days, 400 days, 600 days, mature cow, carcass, and milk, as well as retail beef yield (%), eye muscle area, rib fat, rump fat, and intramuscular fat (IMF). Each trait’s EBV is briefly described in the Supplementary File S1 based on the BREEDPLAN descriptions.

### 2.2. Phenotypic and EBVs Data

The BREEDPLAN database (https://breedplan.une.edu.au/search-login/, accessed in January and May 2020) archives information on registered cattle of several breeds including on-farm records and the genetic evaluation. Each animal has recorded information on head status (Horn, Scur, or Poll based on phenotype), birth date, parents, and EBVs of several traits in categories such as growth (weight), fertility, carcass, behavior, and feed efficiency. A total of 2,467,000 records of animals across eight Australian breeds with known head status were acquired from the BREEDPLAN database (Table 1). Following preliminary assessment and quality control for removing incomplete data (e.g., young animals with low accuracies), trait EBVs and their accuracy were obtained for 1,663,201 animals. All animals kept for analyses had EBVs, accuracies, and a defined head status. Efforts were made to ensure a mix of animals from across different herds registered through the breed societies. BREEDPLAN EBVs are classified for interpreting accuracy [21], such that less than 50% = preliminary, 50–74% = medium, 75–90% = medium–high, and above 90% = high accuracy estimates of the animal’s true breeding value. Note that the accuracy of an EBV increases as more information, such as repeated measures and relatives (pedigree, siblings, and progeny), are included into the prediction models, meaning that more animals have their EBVs within low–medium range than medium–high and high categories. Of the obtained data, 1,248,085 and 421,353 animals had EBVs above 50% and 70% accuracies, respectively (Table 1).

### 2.3. Statistical Analyses

All analyses were performed within-breed for each of the eight breeds. Analyses were generally restricted to animals born between 2000 and 2018 because genetic bases of selected breeds were established before 2000 and poll breeding and trait estimates were initiated. However, animals born before 2000 were grouped to provide a historical genetic base of each breed. All analyses were performed as pair-wise comparisons between two cohorts, polled and horned. Phenotypically scurred animals were excluded to avoid any bias due to head status misclassification. All analyses were performed with consideration of the levels of EBV accuracies as well as the number of animals with EBVs above a particular accuracy threshold.

The filtered and structured dataset analyses were performed by using the R program [23] to compute the statistics for each trait of a breed, and were analyzed for the poll vs. horn cohorts. Summary statistics of Mean (95% confidence intervals) and Standard Deviation (SD) were computed for the two cohorts (horned and polled). Descriptive statistics for pair-wise comparisons between the means were performed by the t-tests with pooled SD, and *p*-values were obtained by using the *t.test* function in R-package “*stats*”. Effect sizes (ES) on each trait due to polledness were computed using the Cohen’s *d* [24,25]: $d=\frac{\mathrm{Mean}\;\mathrm{of}\;\mathrm{polled}-\mathrm{Mean}\;\mathrm{of}\;\mathrm{horned}}{Pooled\;SD}$, where $Pooled\;SD=\sqrt{\frac{{\left(\mathrm{SD}\;\mathrm{of}\;\mathrm{polled}\right)}^{2}+{\left(\mathrm{SD}\;\mathrm{of}\;\mathrm{horned}\right)}^{2}}{2}}$. Moreover, R-package “*psych*” (function: *cohen.d.ci*) was used to compute 95% confidence intervals for ES [26]. Point Biserial Correlations (PBC)—a correlation measure *r* of the strength of association between continuous-level variables (i.e., trait-wise EBVs) and a binary variable of head status (poll, horn)—were computed with *biserial.cor* function in R-package “*ltm*” [27].

Results of these analyses were interpreted as follows: *p*-values were deemed significant above the adjusted α for multiple-comparison by using the Bonferroni correction at α=0.05/n, and *n* is total number of comparisons (analyses), i.e., *n* = *i* × *j*, where *i* is recorded traits with EBVs and *j* is number of breeds. ES (*d*) and PBC (*r*) values provide direction for interpretation of which of the two cohorts are better in a comparison to communicate the practical significance of results. Our analyses were performed such that positive values indicate polled-as-favorable while negative values indicate horned-as-favorable. Two traits (Rib Fat, Rump Fat) can be considered in either direction depending on the breeding objectives. Summary graphs are shown to compare EBVs of horn vs. poll cohorts as Violin-plots using modified functions of R-package “*vioplot*” [28].

## 3. Results

### 3.1. Population Trends of Polledness

Commercial beef producers and feedlots in Australia have seen changing preferences for polled cattle in the past two decades due to increased awareness of the critical importance of improving animal welfare, consumer choices, and costs and risks associated with physical dehorning and disbudding.

**Figure 1:**
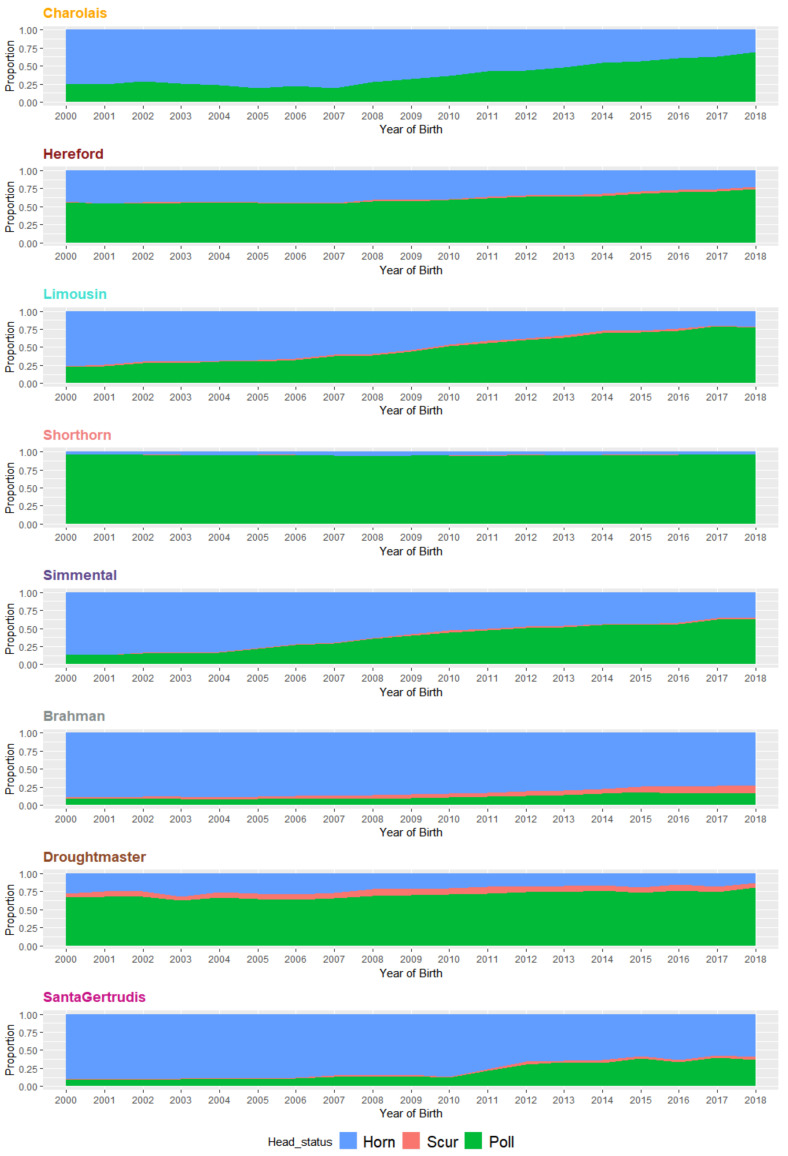
Breed-wise population trends of horn, scur, and poll animals from 2000 to 2018.

The proportion of polled animals in commercial herds have also trended higher during the period (birth years) from 2000 to 2018 across all breeds (Figure 1). Only three breeds (Hereford, Shorthorn, and Droughtmaster) had more than 50% polled animals prior to 2000, and frequencies of polled cattle in these breeds have continued to increase, with greater than 75% by 2018. Conversely, in 2000, the proportion of polledness in five breeds (Charolais, Limousin, Simmental, Brahman, and Santa Gertrudis) was below 25%. Charolais, Limousin, and Simmental have attained substantial increases in polledness to above 60% for their 2018-born cohort. The Brahman and Santa Gertrudis have retained lower poll frequencies at ~20% and 37% respectively, although Santa Gertrudis has trended upwards since 2011.

### 3.2. Accuracy Thresholds of EBVs for Optimal Analyses

Accuracy thresholds are important considerations for comparing genetic merit between polled and horned types across the eight breeds. The accuracy of the BREEDPLAN EBVs ranged from 20% to above 90% (Supplementary Figure S1). As the accuracy thresholds increased beyond 50%, the sample size (N) for several breeds started to rapidly decrease for one or both head types, which limited statistical analyses. While using EBVs with higher accuracies is intuitively a better comparison, smaller sample sizes are an inevitable consequence. Therefore, statistical analyses were performed at two levels of EBVs’ accuracy: 50% and 70%. The initial analyses were performed using 50% accuracy thresholds to account for optimum sample size in each breed. Then, 70% accuracy levels were used to validate those finding relying on presumably influential animals given they had extensive progeny and pedigree records to acquire the higher accuracy of EBVs.

### 3.3. Comparison between EBVs of Beef Production and Head Status

**Figure 2:**
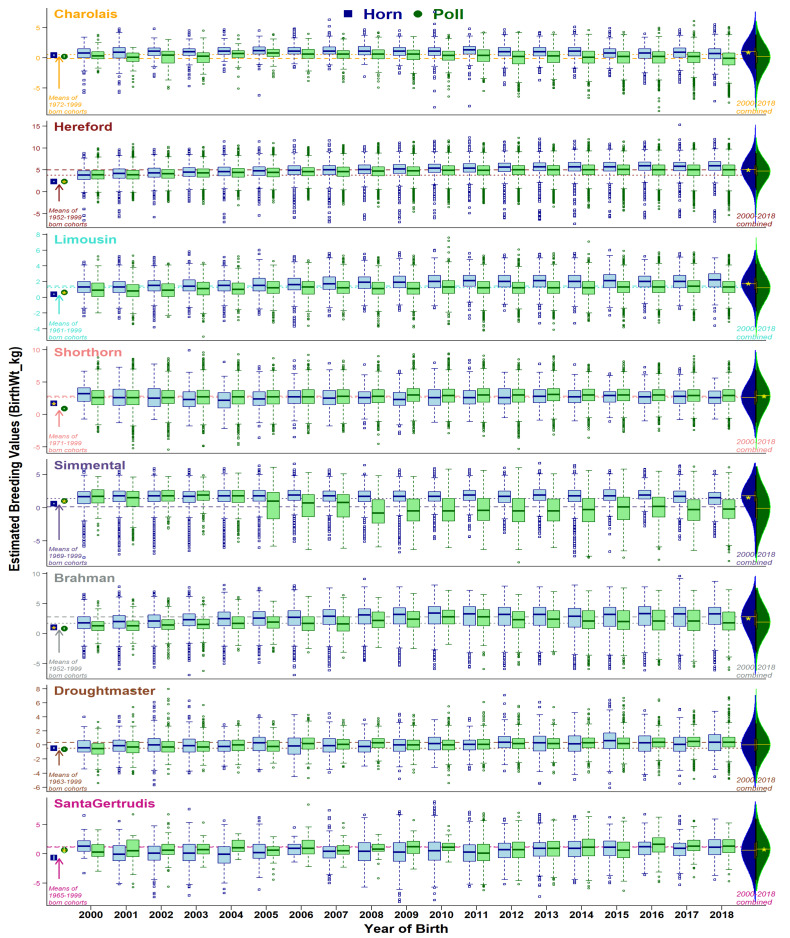
Boxplots of birth weight EBVs (accuracy ≥ 50%) for horn (blue) and poll (green) cohorts born from 2000 to 2018. Dotted and dashed lines show breed averages at the start (2000) and end (2018) of the selected period. Points at the left side of graphs show cohort averages for recorded animals born before 2000. Violin plots on the right side show the distribution of horn and poll cohorts during 2000 to 2018 combined, with a yellow star representing a statistical significance of mean differences.

**Figure 3:**
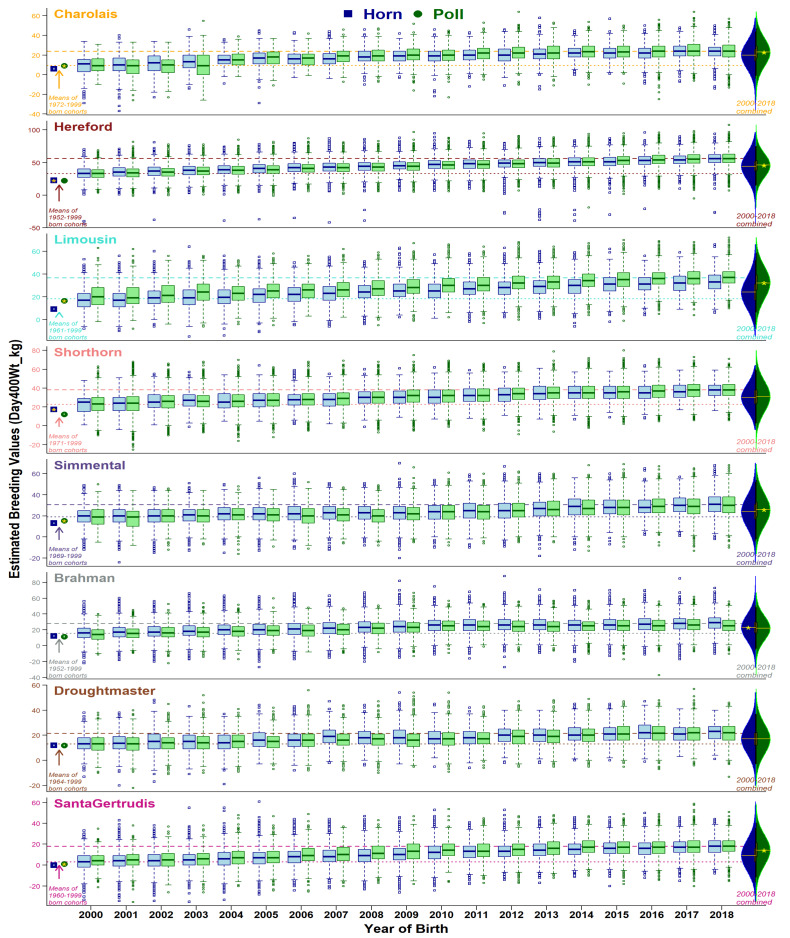
Boxplots of 400 day weight EBVs (accuracy ≥ 50%) for horn (blue) and poll (green) cohorts born from 2000 to 2018. Dotted and dashed lines show breed averages at the start (2000) and end (2018) of the selected period. Points at the left side of graphs show cohort averages for recorded animals born before 2000. On the right-side, violin plots show the distribution of horn and poll cohorts during 2000 to 2018 combined, with a yellow star representing a statistical significance of mean differences.

**Figure 4:**
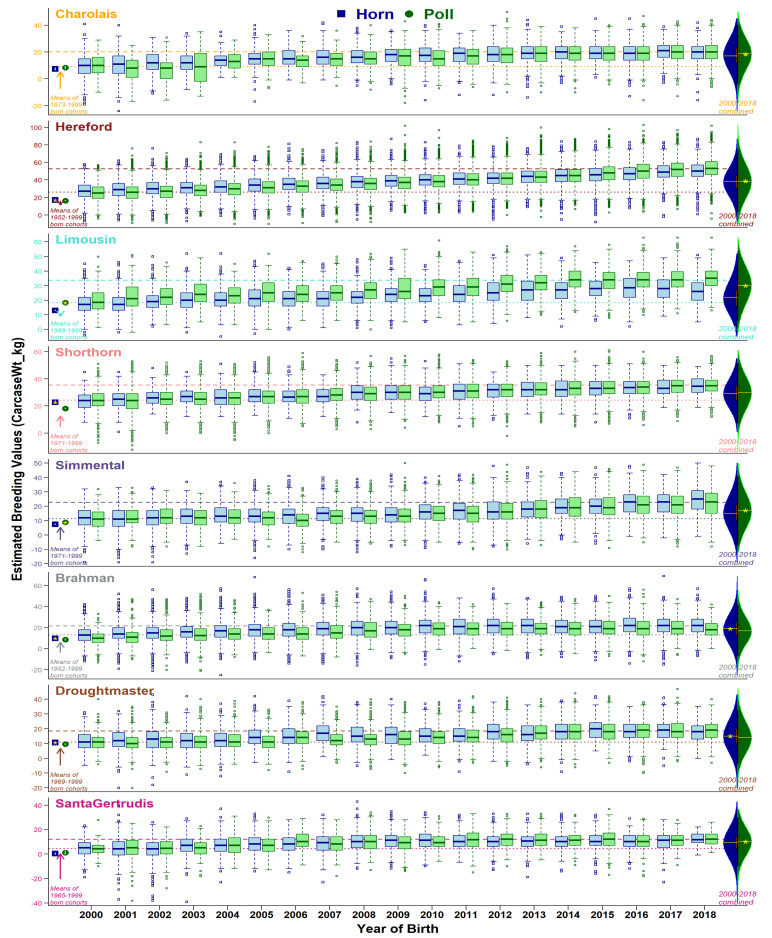
Boxplots of carcass weight EBVs (accuracy ≥ 50%) for horn (blue) and poll (green) cohorts born from 2000 to 2018. Dotted and dashed lines show breed averages at the start (2000) and end (2018) of the selected period. Points at the left side of graphs show cohort averages for recorded animals born before 2000. On the right side, violin plots show the distribution of horn and poll cohorts during 2000 to 2018 combined, with a yellow star representing a statistical significance of mean differences.

**Figure 5:**
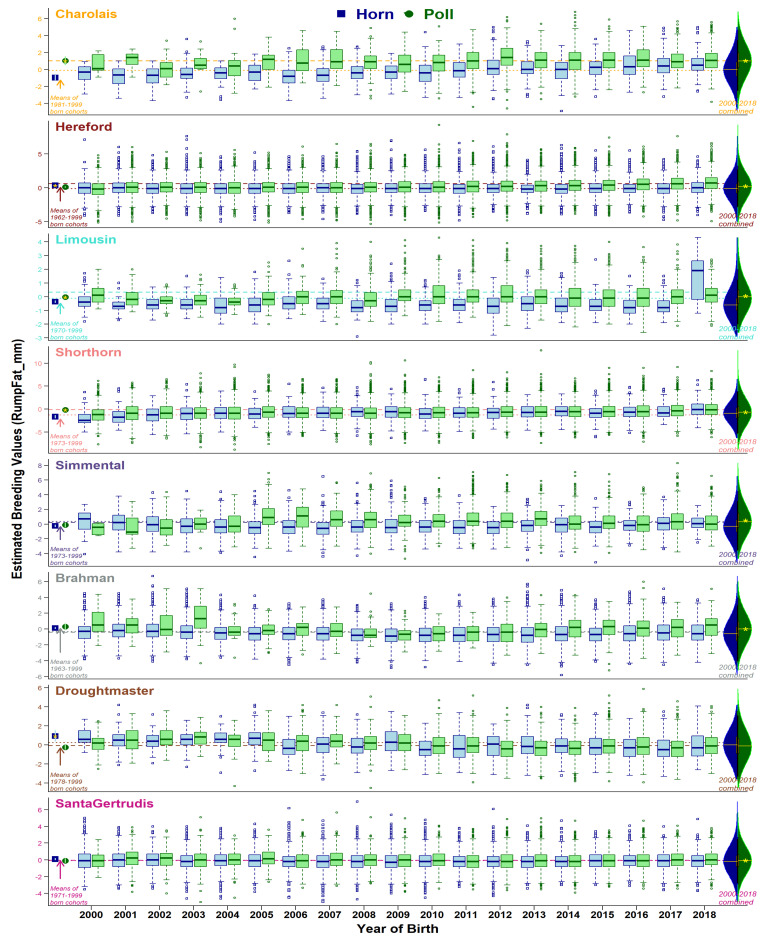
Boxplots of rump fat EBVs (accuracy ≥ 50%) for horn (blue) and poll (green) cohorts born from 2000 to 2018. Dotted and dashed lines show breed averages at the start (2000) and end (2018) of the selected period. Points at the left side of graphs show cohort averages for recorded animals born before 2000. On the right side, violin plots show the distribution of horn and poll cohorts during 2000 to 2018 combined, with a yellow star representing a statistical significance of mean differences.

Comparison of horn and poll population distributions of EBVs are presented from three perspectives: (i) historical status (animals born before year 2000), (ii) yearly changes from 2000 to 2018, and (iii) 2000–2018 combined. Ongoing selective breeding for or against the set of traits has modulated the production dynamics of the beef industry. As expected, overall genetic merit for recorded traits in the eight breeds studied have experienced noteworthy changes within 2000–2018 (see overall changes in Supplementary Table S2, and yearly trend-lines for breed-average in Figure 2, Figure 3, Figure 4 and Figure 5, and Supplementary Figures S2–S9). Trait-wise EBVs distributions of intra-breed polled vs. horned types provide insights (Supplementary Table S1) about the practical outcomes and statistical significance (*t*-tests) of producing polled cattle, as shown in the following sections.

#### 3.3.1. Birth Weight

A comparison of the historical cohorts’ EBVs showed three patterns: (i) non-significant differences between the birth-weight means in Charolais and Droughtmaster, (ii) polled cattle were significantly (Bonferroni-corrected *p* = 0.05) higher than horned in Hereford, Limousin, Simmental, and Santa Gertrudis, and (iii) horned animals had significantly higher weights at birth than their polled contemporaries in Shorthorn and Brahman (Figure 2). Generally, annual breed-averages of birth-weight EBVs were increasing marginally from 2000 to 2018, except in Charolais, Simmental, and Santa Gertrudis. Interestingly, within-cohort annual trends showed significantly changes in the birth weights of five European breeds. Their combined means (2000–2018) of polled and horned types were found to be the opposite to their historic patterns in Charolais, Hereford, Limousin, Shorthorn, and Simmental. However, the relative advantage for birth weights between both head types remained unchanged in the Zebu (Brahman) and Composite (Droughtmaster and Santa Gertrudis) breeds.

In beef production systems, birth weight is an important economic trait with moderate heritability. The selection for a low or high EBV depends on multiple associated traits, including calving ease, growth rate, and adult weights. Larger birth weights are associated with calving difficulty, which can be a major source of financial loss due to additional costs for calving assistance, loss of calf, and occasional heifer death. On the other hand, lower weights at birth are unfavorably correlated with growth and weight traits during later life stages. Therefore, animals with average birth weights can be ideal for breeding to avoid any negative impact on farm productivity. Thus, higher average EBVs of birth weights in the horned cohorts of five breeds (Charolais, Hereford, Limousin, Simmental, and Brahman) may be a disadvantage. Instead, the distribution of birth weights of polled cohorts in Shorthorn, Droughtmaster, and Santa Gertrudis showed an advantage of relatively small magnitude over their horned herd-mates. Overall, polled cohorts presented higher genetic merit for birth weight.

#### 3.3.2. Weights at 200, 400, and 600 Days

Weight traits measured at different life stages (200, 400, and 600 days) showed essentially similar trends across all breeds (Figure 3, Supplementary Figures S2 and S3). Historically, Charolais, Limousin, Simmental, and Santa Gertrudis were significantly better for polled types, while Hereford, Shorthorn, Brahman, and Droughtmaster (only at 600 days) had better genetic merit of their horned cohorts. From 2000 to 2018, all breeds have shown uniformly upward trends of EBVs for the three traits at variable rates. Interestingly, within two decades, the rate of genetic gain for polled animals’ weight was relatively higher for most breeds, most notably in Hereford and Shorthorn. There seems to be a selective advantage to using polled animals. Brahman remained the exception, with horned animals performing better for all three traits over the period examined.

#### 3.3.3. Mature Cow and Carcass Weights

EBVs of mature cow (Figure S4) and carcass (Figure 4) weights have also shown an overall increase in the genetic potential of all eight breeds but contradicted some trends of earlier growth traits (e.g., birth weight) for polled and horned cattle. Pre-2000-born cohorts showed similar mature cow EBVs in four breeds (Charolais, Simmental, Droughtmaster, and Santa Gertrudis), higher EBVs for horned in two breeds (Shorthorn and Brahman), and higher EBVs of polled in two breeds (Hereford and Limousin). Carcass weight EBVs were similar for polled and horned cohorts in Charolais and Santa Gertrudis, higher in polled Limousin, and higher in horned Simmental and Brahman. In general, selective breeding for polledness over the two decades has shown genetic improvements for both traits. As a result, post-2000-born animals’ EBVs are favorable in polled animals, except for Charolais, Simmental, and Brahman for mature cow weight, and Brahman and Droughtmaster for carcass weight.

#### 3.3.4. Milk

EBVs of milk—indicators of maternal effect on calf weaning (200 days) weight—were historically higher in horned cohorts of five breeds (Hereford, Shorthorn, Simmental, Brahman, and Droughtmaster) as compared to polled in two breeds (Charolais and Limousin) (Supplementary Figure S5). Four breeds (Charolais, Hereford, Limousin, and Droughtmaster) showed increasing trends of breed averages. Three breeds (Shorthorn, Simmental, and Santa Gertrudis) remained stable, while Brahman experienced a fall in maternal effects on 200-day weight (milk EBVs) during 2000–2018. Horn and poll cohorts ranked consistently between pre- and post-2000-born populations, except Shorthorn and Brahman, that showed higher EBVs in polled cohorts when compared to their leading horned animals.

#### 3.3.5. Retail Beef Yield

Retail beef yield EBVs (Supplementary Figure S6) has surprisingly shown only horned cohorts to be superior in historically three (Charolais, Limousin, and Shorthorn) and recently five (Charolais, Hereford, Limousin, Shorthorn, and Simmental) breeds. In most cases, the breed averages remained unchanged during the two past decades, except for some constricted trends of decrease (Charolais and Droughtmaster) and increase (Brahman and Santa Gertrudis). Note that some annual EBVs distribution of the Brahman and Droughtmaster may have been affected by limited sample sizes of horned and polled cohorts (Supplementary Table S1).

#### 3.3.6. Eye Muscle Area

EBVs of eye muscle area (Supplementary Figure S7) were also historically better for horned cohorts in only three breeds (Charolais, Limousin, and Shorthorn), while horned and polled types were alike in other breeds. However, Breed-averages constantly increased between 2000 and 2018 across all breeds. Besides, poll cohorts were significantly superior in five breeds (Hereford, Limousin, Simmental, Droughtmaster, and Santa Gertrudis) against the horned cohorts, which showed significant superiority in other breeds (Charolais, Shorthorn, and Brahman).

#### 3.3.7. Intra-Muscular, Rib, and Rump Fat

Three traits of body fat deposition in intra-muscles (Supplementary Figure S8), rib (Supplementary Figure S9), and rump (Figure 5) areas showed a persistence for polledness with slow incremental rates. Historically, horned types were significantly higher in only Hereford (intra-muscular and rump fat) and Droughtmaster (rump fat). From 2000 to 2018, all breeds, but Brahman (intra-muscular and rib fat) and Droughtmaster (rump fat), have shown increasing mean EBVs of fat traits. Consequently, poll types have become significantly superior for three body fat traits in all breeds with one exception of intra-muscular fat in Santa Gertrudis.

### 3.4. Genetic Trends of Birth Weight Contrast Subsequent Weight Traits

**Figure 6:**
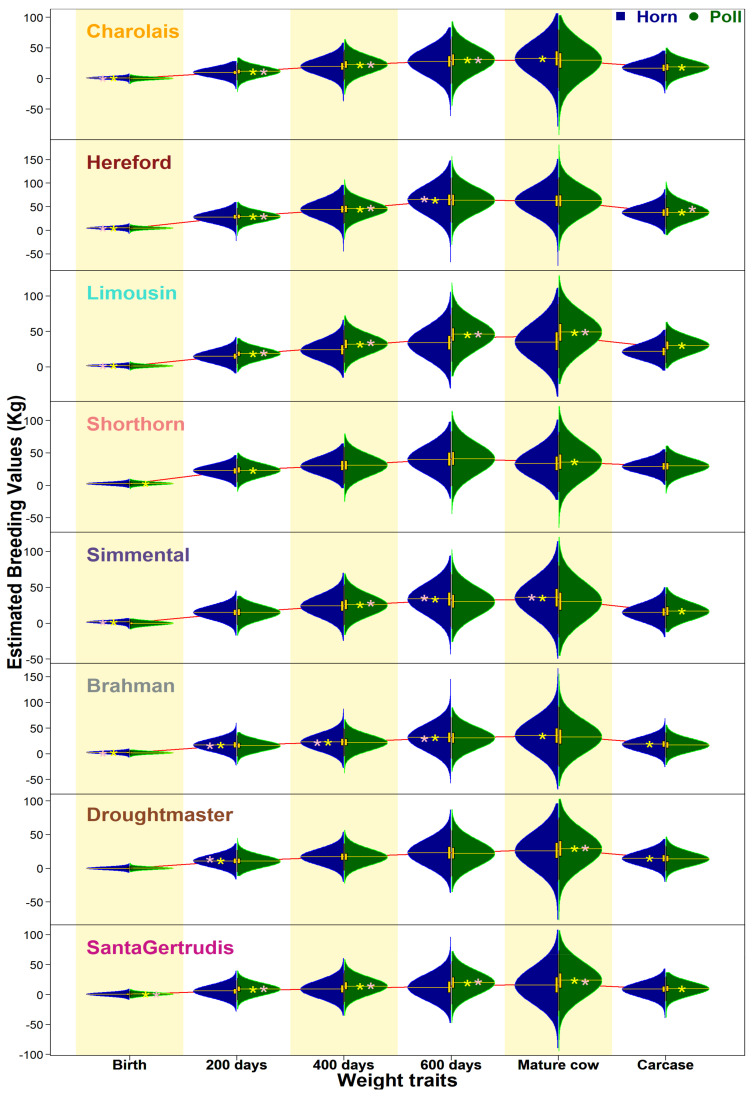
Violin plots of BREEDPLAN EBVs (accuracy ≥ 50%) of weight traits at different life stages in eight breeds comparing horn and poll cohorts born 2000–2018. Yellow and pink steric represent a statistically significant higher cohort-mean at 50% and 70% accuracies of EBVs, respectively.

Earlier results noted a contrasting pattern of EBVs between birth weights as compared to other weight traits at the next life stages (200 days, 400 days, 600 days, mature cow and carcass weights). Figure 6 shows a summary comparison of weight traits in eight breeds using EBVs (2000–2018) at 50% accuracy and re-assessed their significance at 70% accuracy. Out of eight, five breeds (Charolais, Hereford, Limousin, Simmental, and Brahman) had significantly higher EBVs for birth weights of the horned cohorts. Interestingly, the polled cohorts of four of them (except Brahman) showed higher genetic merit at 200- and 400-day weights at both 50% and 70% accuracy of EBVs. In fact, Brahman was the only breed to have horned cohorts performing as genetically superior throughout; however, their mature cow and carcass weights were not found significantly different using EBVs at 70% accuracy thresholds. On the other hand, polled cohorts of Santa Gertrudis significantly excelled for all live weights (accuracy = 50% and 70%) and carcass weight (50%). As another example, Limousin (horned superior) contrasts Santa Gertrudis (polled superior) at birth; however, polled animals of both breeds equally achieved superior genetic estimates of subsequent traits. Nonetheless, polled types of most beef breeds have enhanced genetic merit for postnatal growth and carcass weight (Figure 6 and Supplementary Figure S10).

### 3.5. Effect Size of Polledness on Beef Production and Quality Traits

**Figure 7:**
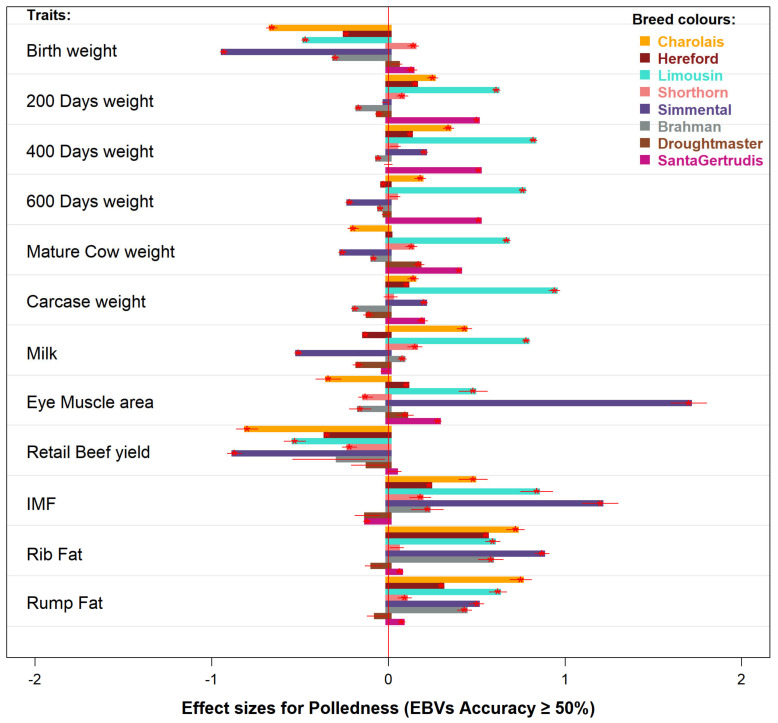
Effect sizes (Cohen’s *d*) of polledness in 8 breeds using BREEDPLAN EBVs (accuracy ≥ 50%) on 12 production traits. Red lines within each bar show 95% confidence intervals of effect sizes. Statistically significant results are shown with a red star. An effect size indicates how much the two cohort means differ by a *d* standard deviation. A positive value indicates higher genetic merit for polled cattle.

Practical implications of polledness are shown by its effect size (Cohen’s *d*) on a particular trait (Figure 7). Out of 12 traits investigated here, polledness has a positive effect size ranging from very small (0.01) to medium (0.5) in the majority of breeds (Supplementary Table S1). In contrast, two traits (birth weight and retail beef yield) commonly have negative *d* values for polled animals. Lighter birth weights are generally considered favorable for calving ease, and data suggests polled animals will achieve similar or better growth rates thereafter. A negative correlation of polled with retail beef yield was unexpected and will require further investigation, especially in Charolais (−0.80) and Simmental (−0.87). However, substantially low-spectrum EBVs of retail beef yield (%) have been noted. The magnitude of difference between the polled and horned cohorts has reduced in recent five years (Supplementary Figure S6) such that undesirable effects of polledness in current breeding populations are unlikely.

Brahman, which earlier showed poll types consistently lagging behind the horned animals for growth traits, were found to have relatively very small effect sizes of polledness on all those traits (Supplementary Table S1). Genetic merit of poll animals for the weights at birth (*d* = −0.30), 200 days (−0.19), 400 days (−0.06), 600 days (−0.05), mature cow (−0.08), and carcass (−0.19) will have limited practical implications on beef production of Brahman herds. Furthermore, using EBVs with higher accuracy (70%) demonstrated a further drop in significance occurrences and constricted the effect sizes of polledness on target traits in most breeds, although a few opposite trends were seen, probably impacted by lower sample sizes (Supplementary Figure S11 and Table S1).

## 4. Discussion

As a consequence of increasing concerns about animal welfare and the costs of bruising and dehorning [4], there has been sustained selection for polled cattle. Historical examination of breed-wise EBVs (accuracy 50%) for a range of production and carcass quality traits indicated that polled and horned genetic effects highly varied for these traits. However, it was observed that as the accuracy of EBVs increased to 70%, many of the significance differences were not sustained. The results are consistent with previous research on the impact of polledness on beef productivity of different breeds of cattle [5,14,16,29].

The magnitude of an occurrence is quantitatively measured by its effect size. The larger the effect size, the stronger the relationship between two variables, e.g., polledness and birth weight. The effect sizes (Cohen’s *d*) and correlations (Point Biserial or Pearson’s *r*) of poll status with the beef traits investigated here were consistently in the small range (*d* < 0.2 and *r* < 0.1), such that statistically significant differences will have very small practical significances. Mostly, the trends of trait-wise EBVs differences and their effect sizes were neither consistent nor unidirectional across the eight breeds, hence genetics controlling the head status is not deemed directly causal for differences observed.

Interestingly, a promising observation in favor of poll breeding was that the polled cohorts that had lower EBVs at early age (birth weight) showed above average EBVs for later growth and weight (200 days, 400 days, 600 days, mature cow, and carcass) traits. In later life, poll types became as good as or better than their horned contemporaries, which was also consistent with previous findings using live weights [14]. Some contrasting observations were seen in Brahman polled cohorts consistently showing undesirable production parameters for live weight and carcass traits (Figure 6). However, the effect size (*d*) and correlation (*r*) with polledness were very small for EBVs of weight at birth (*d* = −0.30, *r* = −0.08), 200 days (−0.19, −0.05), 400 days (−0.06, −0.02), 600 days (−0.05, −0.01), and mature cow (−0.08, −0.02) and carcass (−0.19, −0.05) weights. These findings are novel contributions to augment poll breeding across the beef industry for sustainable production and objectively better animal welfare.

Genetic gain for different traits within each breed remains variable (Figure 2, Figure 3, Figure 4 and Figure 5, Supplementary Figures S2–S9), explained by the discordant relationships between various pairs of BREEDPLAN EBVs. For example, high and positive correlations among live and carcass weights showed consistently increasing breed-average trends of genetic merits [30]. On the other hand, highly positive correlation amongst the body fat traits yet negative associations to weight traits lead to a slower rate of genetic gain [31]. Furthermore, two traits of subcutaneous fat deposition (Rib fat, Rump fat) can be considered favorable depending on the breeding objectives of a herd/breed. Obviously, weight traits are major contributors to economic returns and generally positive statistics show an overall favorable effect of polledness within each breed.

Despite the increasing trends in all breeds, poll gene frequency remains low in the tropically adapted Brahman and Santa Gertrudis. Hence, loss of genetic diversity and consequently elevated levels of inbreeding may occur in the progeny of closely related polled animals. Inbreeding depression in production traits, as well as the potential increase of detrimental allelic homozygosity [32] and associated diseases (e.g., Pompes), are risks [33,34]. Generally, in livestock species, a 0.137% decrease of the mean of a trait has been estimated for every unit (1%) increase in inbreeding and the loss was larger (0.351%) for production traits [34]: Zebu cattle decreased by 0.269% for weight traits [35]. In beef cattle, inbred animals have consistently shown an adverse effect on growth traits from birth to maturity. For example, a 1% increase in inbreeding coefficient was associated with 0.514 kg decreased birth weights in Brahman [36], and 1.20 kg weaning and 2.03 kg yearling weights in Hereford [37]. Therefore, care must be taken to purge high levels of relatedness and inbreeding depression in the respective breeding programs until there are larger numbers of polled sires and dams. For instance, a gradual increase in poll population of Brahman can prevent inbreeding impacts and maintain a steady rise in genetic gains [38]. Recently, gene-editing technologies have been proven as pertinent tools—depending upon federal regulations—to maintain genetic diversity and rapidly introgress the poll gene into the herds, which are otherwise vulnerable to inbreeding depression [38,39,40].

An eminent challenge is the accuracy of head status phenotyping and subsequent recording in the BREEDPLAN database [41]. New genetic diagnostic tools can eliminate the impacts of phenotyping bias [42]. Previously, we noted that the highest discordance between the phenotype and genotypes were observed in scurred animals (i.e., 19.9% of the scurred samples had homozygous horn alleles, HH [42]), thus they were excluded from these analyses. On the other hand, 10.2% of horned samples carried the polled alleles (9.8% HP and 0.4% PP). The previous study was designed to include an excess of heterozygous animals to optimize the genetic testing assays. A subsequent larger sampling showed that 2.42% of the horned animals were non-concordant (HP and PP). This suggests that some proportion of the horned cohorts were genetically heterozygous at the poll locus, thus the results of this study may have been slightly influenced. Although horned and polled phenotypes were found inaccurately recorded in some breeds, the lower inaccuracy levels (<5%) may not have significantly exacerbated our analyses. It is noteworthy to mention that the polled animals can either be genetically homozygous PP (~53%) or heterozygous HP (~47%). Though P is a dominant allele, genetic evaluations of the polled cohorts of this study are more likely biased due to genome-wide heterozygosity of the HP animals. Future investigations are warranted by using genotype–phenotype concordant head status to account for the perceived bias. Nonetheless, breeding high genetic merit polled animals has become a realistic perspective with high-throughput genetic tools being used for genomic selection and precision breeding [40]. As the poll gene testing and high-density genotyping are being widely implemented into commercial cattle, a larger proportion of genomic evaluated breeding animals will become available.

## 5. Conclusions

Historically, for the eight breeds examined in this study, the frequency of polled cattle was lower, and their genetic merit was variable and often lower than that for horned cattle. Growing awareness about animal welfare and non-invasive alternatives concerning dehorning practices has surged the progress of poll breeding in recent years. Currently, the trends of 12 traits have shown that polledness has no detrimental effects on beef production and meat quality, and indeed, for many traits, polled are statistically superior. However, the effect size in all cases is relatively small (Cohen’s *d* < 0.2), such that there would be no significant genetic gains for evaluated traits through selection of polled animals over horned animals, and vice versa. This study disputed earlier perceptions and provided novel contributions to promote poll breeding in beef cattle for increased sustainability and lowered animal welfare risks. Nevertheless, the potential risks of inbreeding depression should be carefully managed as the proportion of polled animals remains low in Brahman and Santa Gertrudis. Genomic evaluation and gene-editing tools can potentially augment genetic gains while conserving genetic diversity.

## Figures and Tables

**Table 1:** Number of animals with known head status and estimated breeding values (EBVs) data of eight breeds.

Breeds	Head Status	Estimated Breeding Values (EBVs)		
		All *	Accuracy ≥ 50%	Accuracy ≥ 70%
Charolais	43,926	39,853	23,770	9694
Hereford	835,402	668,386	526,841	200,226
Limousin	129,760	133,290	91,179	20,116
Shorthorn	173,777	140,934	122,762	53,292
Simmental	125,478	122,907	88,047	26,284
Brahman	535,005	295,160	210,481	84,793
Droughtmaster	264,967	112,224	55,167	3518
Santa Gertrudis	358,470	150,447	129,838	23,430
**Total**	**2,466,785**	**1,663,201**	**1,248,085**	**421,353**

* Number of animals with EBVs available for at least one of the 12 traits. Some traits were recorded on fewer animals as reported in results (Supplementary Table S1). Data were downloaded from BREEDPLAN between January and May 2020.

## Data Availability

Phenotypic records and estimated breeding values (EBVs) are openly accessible from BREEDPLAN web database of each breed (https://breedplan.une.edu.au/search-login/). The data used in this study were acquired during January and May 2020.

## Supplementary Materials

The following are available online at https://www.mdpi.com/2076-2615/11/3/870/s1, Definition of trait-wise EBVs, Figure S1. Distribution of BREEDPLAN EBVs for growth and beef quality traits above different thresholds of accuracy for cohorts of horn and poll head status in eight breeds of beef cattle, Figure S2. Boxplots of 200-day weight EBVs (accuracy ≥ 50%) for horn and poll cohorts, Figure S3. Boxplots of 600-day weight EBVs (accuracy ≥ 50%) for horn and poll cohorts, Figure S4. Boxplots of mature cow weight EBVs (accuracy ≥ 50%) for horn and poll cohorts, Figure S5. Boxplots of milk EBVs (accuracy ≥ 50%) for horn and poll cohorts, Figure S6. Boxplots of retail beef yield EBVs (accuracy ≥ 50%) for horn and poll cohorts, Figure S7. Boxplots of eye muscle area EBVs (accuracy ≥ 50%) for horn and poll cohorts, Figure S8. Boxplots of intra-muscular-fat EBVs (accuracy ≥ 50%) for horn and poll cohorts born 2000–2018, Figure S9. Boxplots of rib fat EBVs (accuracy ≥ 50%) for horn and poll cohorts, Figure S10. Violin-plots of BREEDPLAN EBVs (accuracy ≥ 70%) of weight traits at different life-stages in eight breeds comparing horn and poll cohorts. Pink steric (*) represent a statistically significant higher mean at 70% accuracies of EBVs, Figure S11. Effect sizes (Cohen’s *d*) of polledness in 8 breeds using BREEDPLAN EBVs (accuracy ≥ 70%) on 12 production traits. Red lines within each bar show 95% confidence intervals of effect sizes. Statistically significant results are shown with a red star, Table S1 Breed-wise sample sizes (N animals) used for the combined, polled, and horned cohorts, their Mean, Standard deviation (SD), standard error of mean (SEM), lower and upper 95% confidence intervals (CI) of mean, T-test, *p*-value, Point biserial correlation, Effect size (ES), and lower and upper 95% CI of ES of 12 traits at 50% and 70% accuracy of EBVs using animals born before and after year 2000. Table S2 Breed-wise overall changes in the EBVs of each trait during 2000 (start) to 2018 (end) at 50% and 70% accuracy.
