# Supplementary material for: Comparison of Genetic Merit for Weight and Meat Traits between the Polled and Horned Cattle in Multiple Beef Breeds
Source: Animals (Basel). 2021 Mar 18;11(3):870. doi: 10.3390/ani11030870 (PMC8003249; doi:10.3390/ani11030870)
Supplement: Supplementary file 1 [file animals-11-00870-s001.zip › animals_Supplementary_Polledenss_Production2/Supplementary_Polledness_Production_Beef_Cattle_FINAL-v2.docx]

**Definition of trait-wise EBVs**

***Birth weight (kg)*:** Weights of calves taken at birth are used to calculate EBVs. Small or moderate birth weight EBVs are more favourable for calving ease because calf birth weight is the biggest genetic contributing factor causing calving difficulty in heifers. On the other hand, small birth weights are also associated with lower overall growth potential. Thus, birth weight and growth need to be carefully balanced for minimum calving difficulty and maximum overall growth.

***200 days weight (kg)***: EBVs are calculated from the weights of calves taken between 80 and 300 days of age. This EBV is a measure of an animal's early growth to weaning and 200 days weight is an important trait for breeders turning off animals as vealers or weaners.

***400 days weight (kg)***: EBVs are estimated from the weights of calves taken between 301 and 500 days of age. The 400 days weight EBV is an important trait for breeders turning off animals as yearlings.

***600 days weight (kg)***: EBVs are calculated from the weights of calves taken between 501 and 900 days of age. The 600 days weight EBV is an important trait for breeders targeting the production of animals suited for heavy weight grass or grain fed markets.

***Mature cow weight (kg)***: EBVs are calculated from weights taken on the cow when her calf’s 200 day (weaning) weight is being measured. Mature cow weight EBVs are an indicator of cow feed requirement and cull cow values. Generally, lighter cows will tend to eat less and consequently have lower feed requirements and be less expensive to maintain. On the other hand, heavier cows may provide higher returns from the sale of cull cows.

***Carcase weight (kg)***: EBVs are estimates based on the hot standard carcase weight (AUSMEAT: www.ausmeat.com.au/WebDocuments/Producer_HAP_Beef_Small.pdf) at 650 days of age. Thus, carcase weight EBV is an indication of the animal’s carcase weight rather than the animal’s yield percentage.

***Milk (kg)***: EBVs are calculated by partitioning the 200 day weight (kg) of calves into growth and milk components. For cows, milk EBVs are estimated for an animal's maternal effect on the 200 day weight of its calf. In the case of sires, this estimates the maternal effect that his daughters will have on the 200 day weight of their progeny. Higher EBVs are beneficial to obtain optimum level of milk production potential among beef cows, although it also depends upon the production system and environment.

***Retail beef yield (%)*:** EBVs are calculated based on boned out retail beef yield in a standard weight steer carcase.

**Eye muscle area (cm^2^):** EBVs are calculated based on eye muscle area at 12/13th rib site in a standard weight steer carcase.

***Rib fat (mm)***: EBVs are estimated based on fat depth at the 12/13th rib site in a standard weight steer carcase.

***Rump fat (mm)***: EBVs are estimated based on fat depth at the P8 rump site in a standard weight steer carcase.

***Intra-muscular fat (IMF, %)***: IMF EBVs are calculated from the intramuscular fat (marbling) at the 12/13 rib site in a standard weight steer carcase.


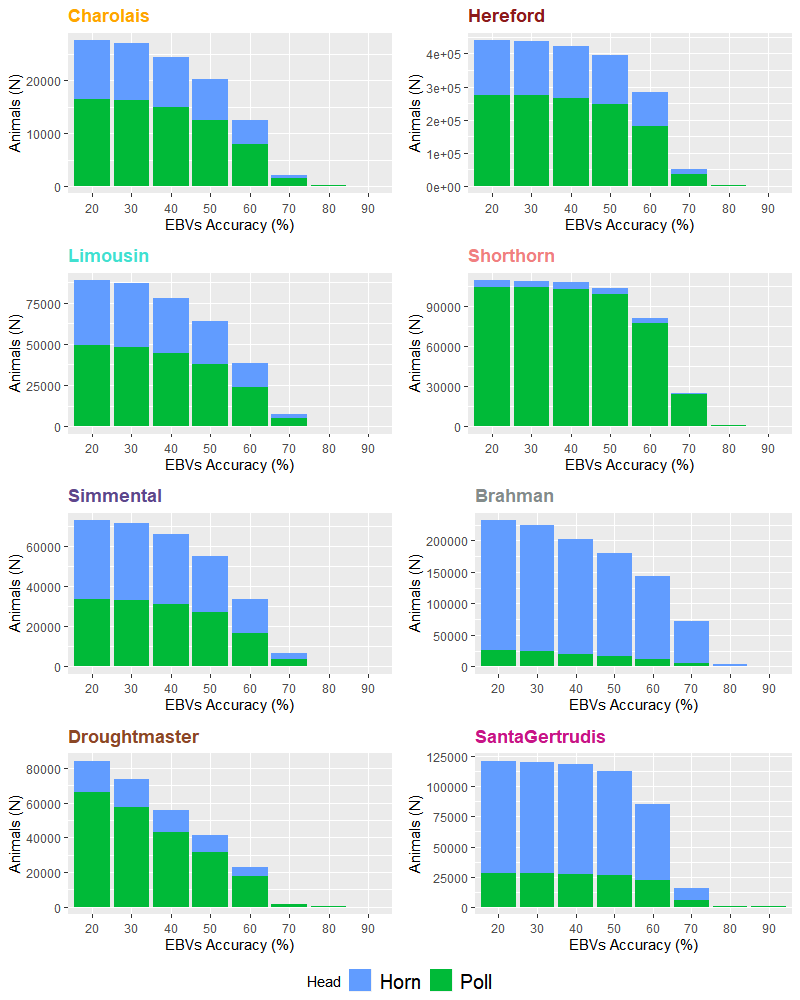


**Figure S1**. Distribution of BREEDPLAN EBVs for growth and beef quality traits above different thresholds of accuracy for cohorts of horn and poll head-status in eight breeds of beef cattle.


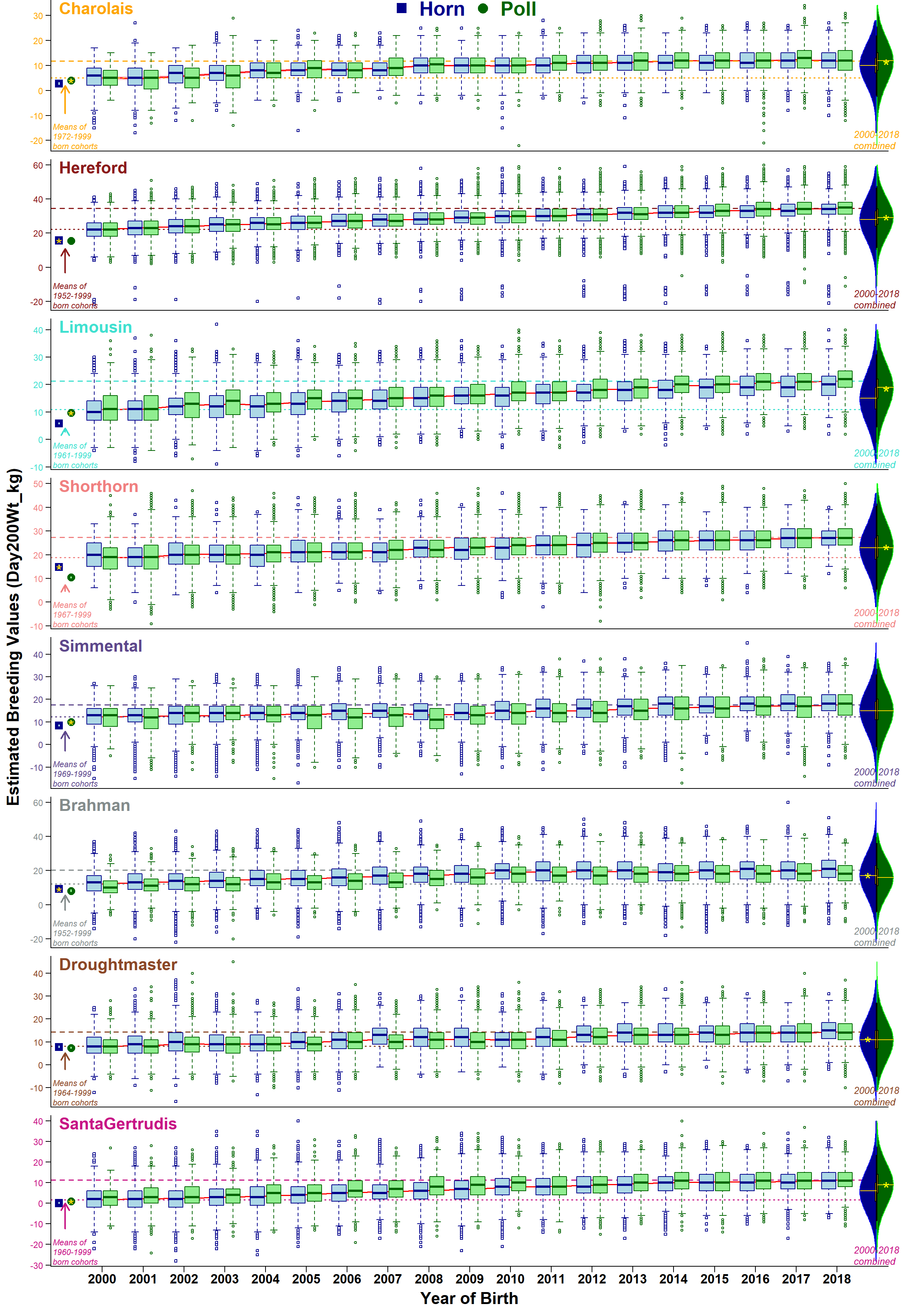


**Figure S2**. Boxplots of 200 days weight EBVs (accuracy ≥ 50%) for horn and poll cohorts.


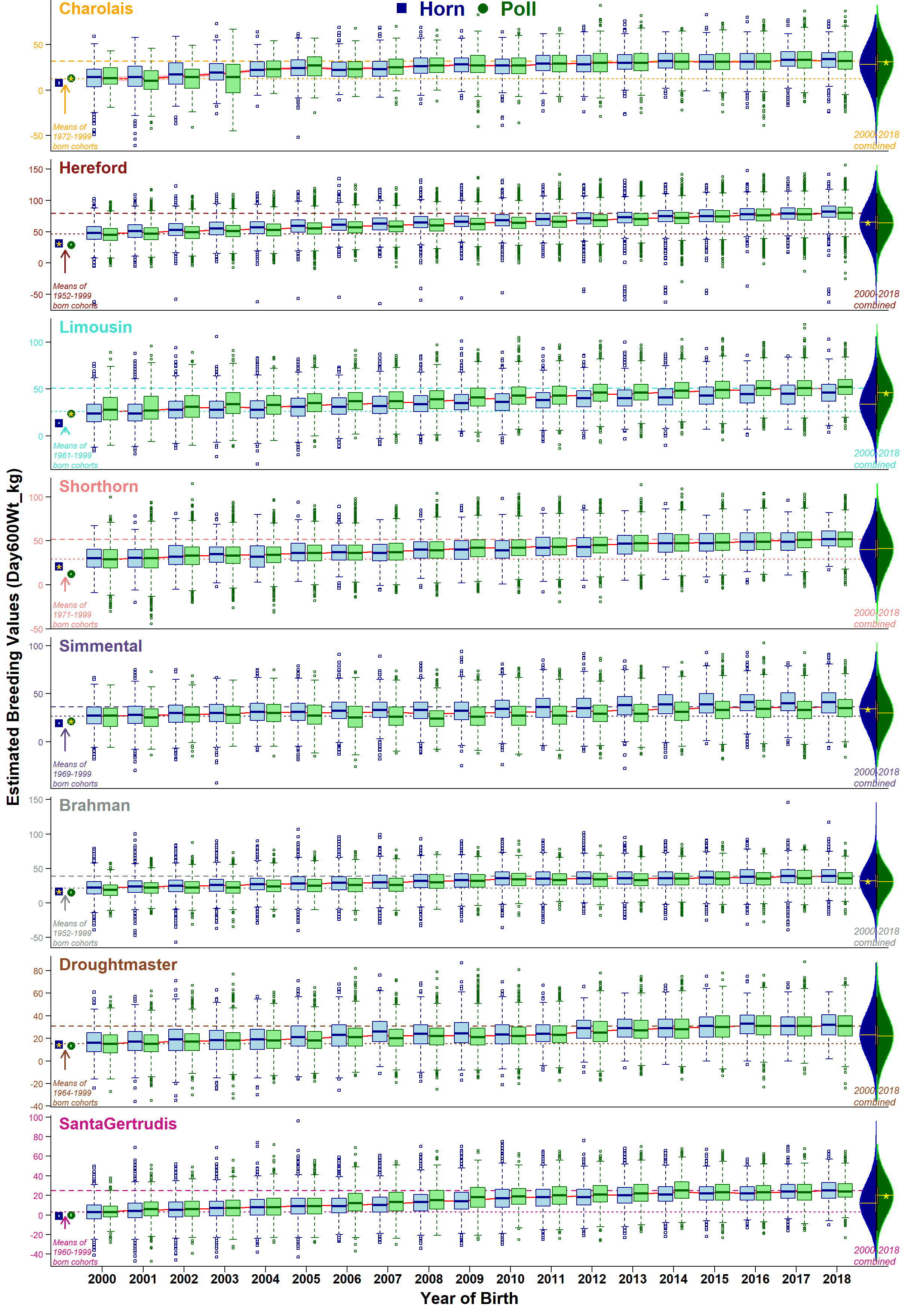


**Figure S3**. Boxplots of 600 days weight EBVs (accuracy ≥ 50%) for horn and poll cohorts.


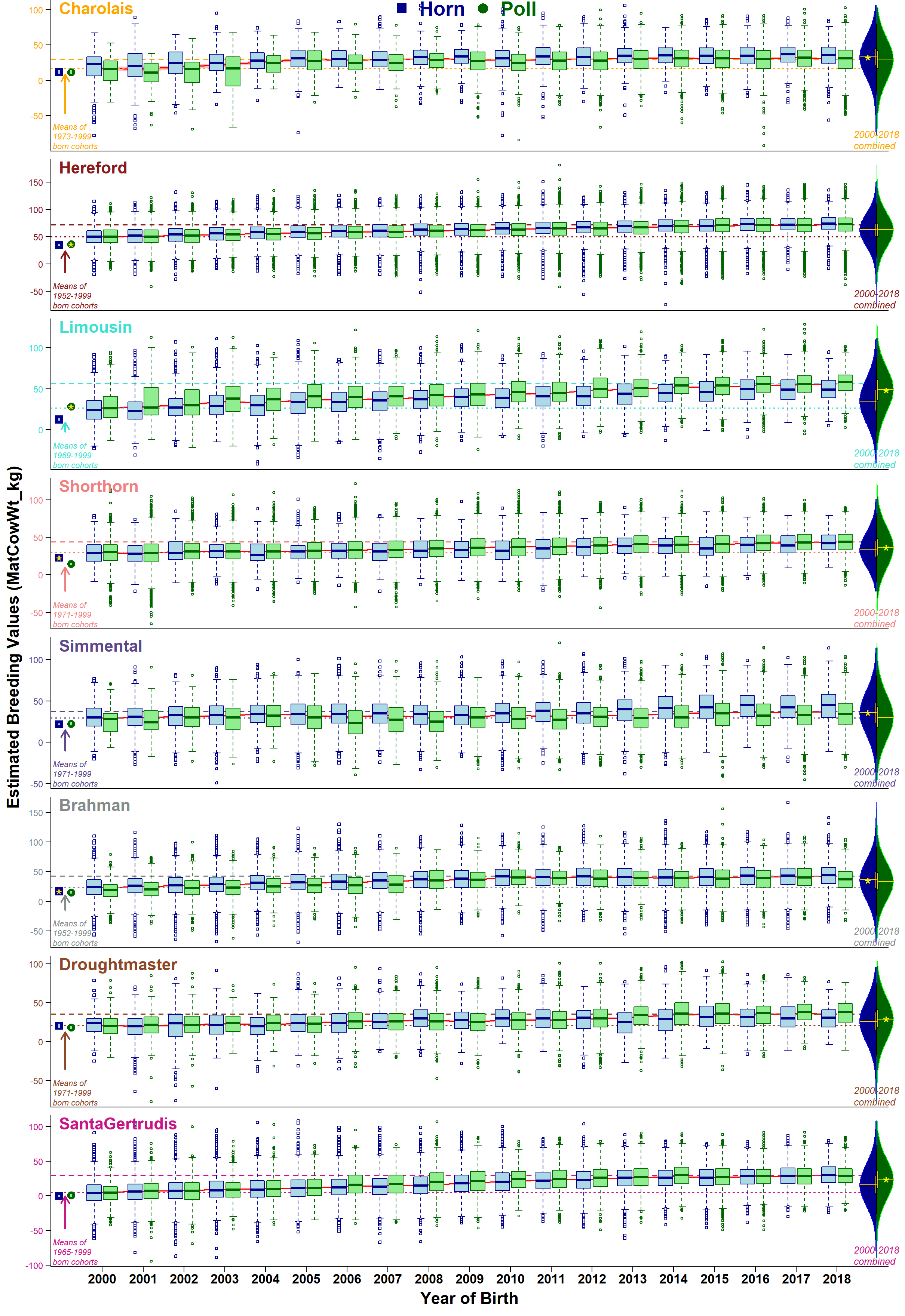


**Figure S4**. Boxplots of mature cow weight EBVs (accuracy ≥ 50%) for horn and poll cohorts.


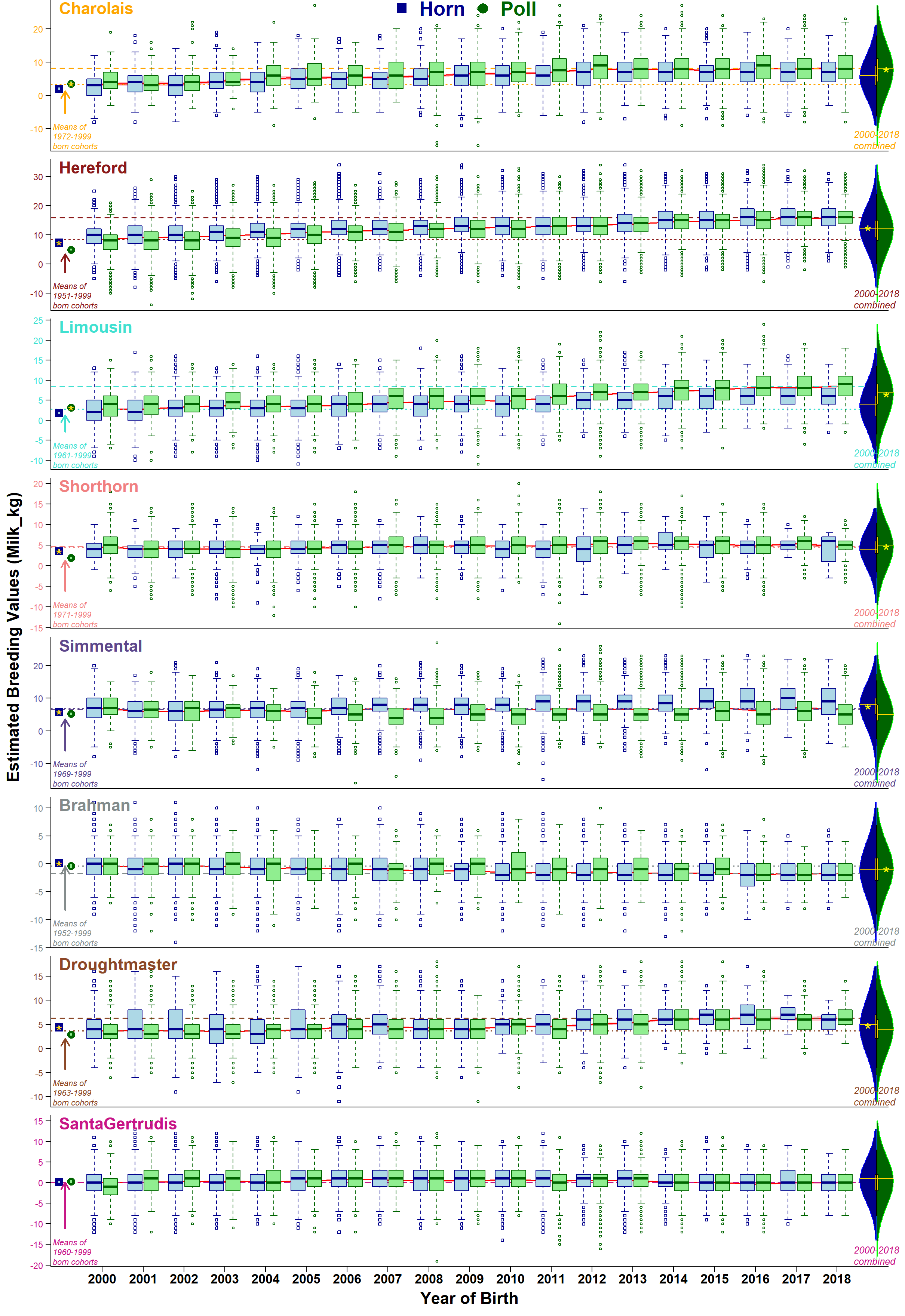


**Figure S5**. Boxplots of milk EBVs (accuracy ≥ 50%) for horn and poll cohorts.


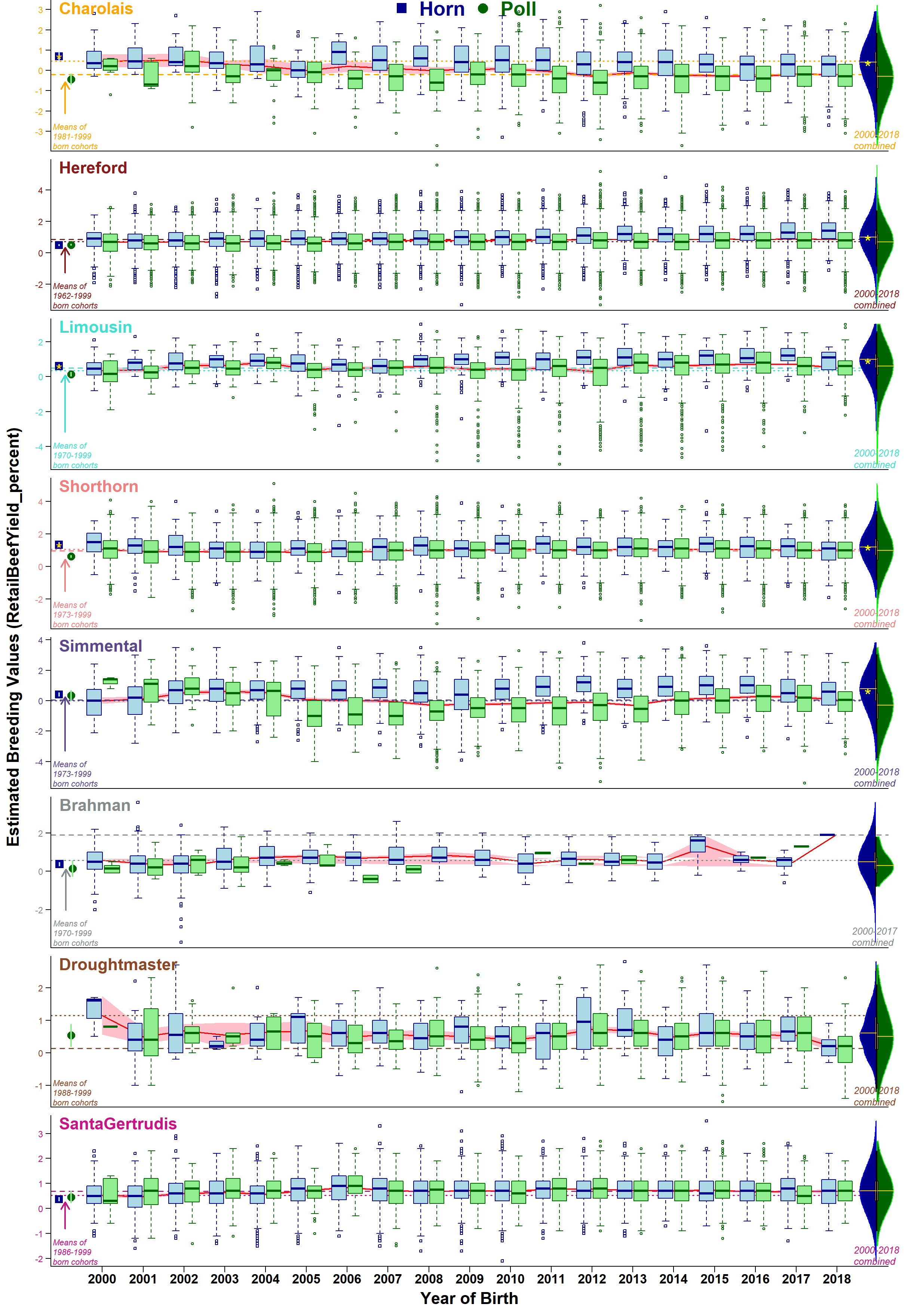


**Figure S6**. Boxplots of retail beef yield EBVs (accuracy ≥ 50%) for horn and poll cohorts.


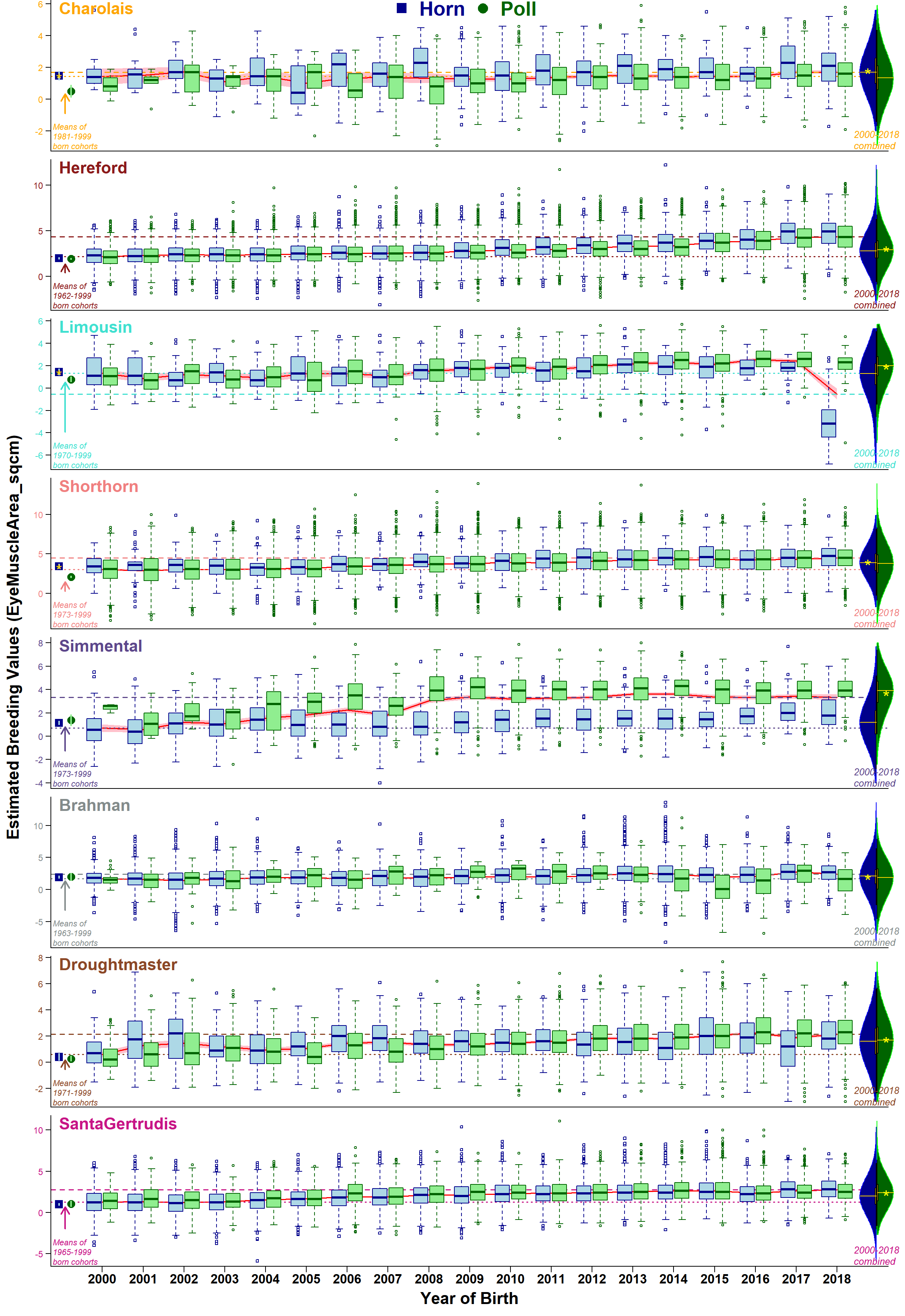


**Figure S7**. Boxplots of eye-muscle-area EBVs (accuracy ≥ 50%) for horn and poll cohorts.


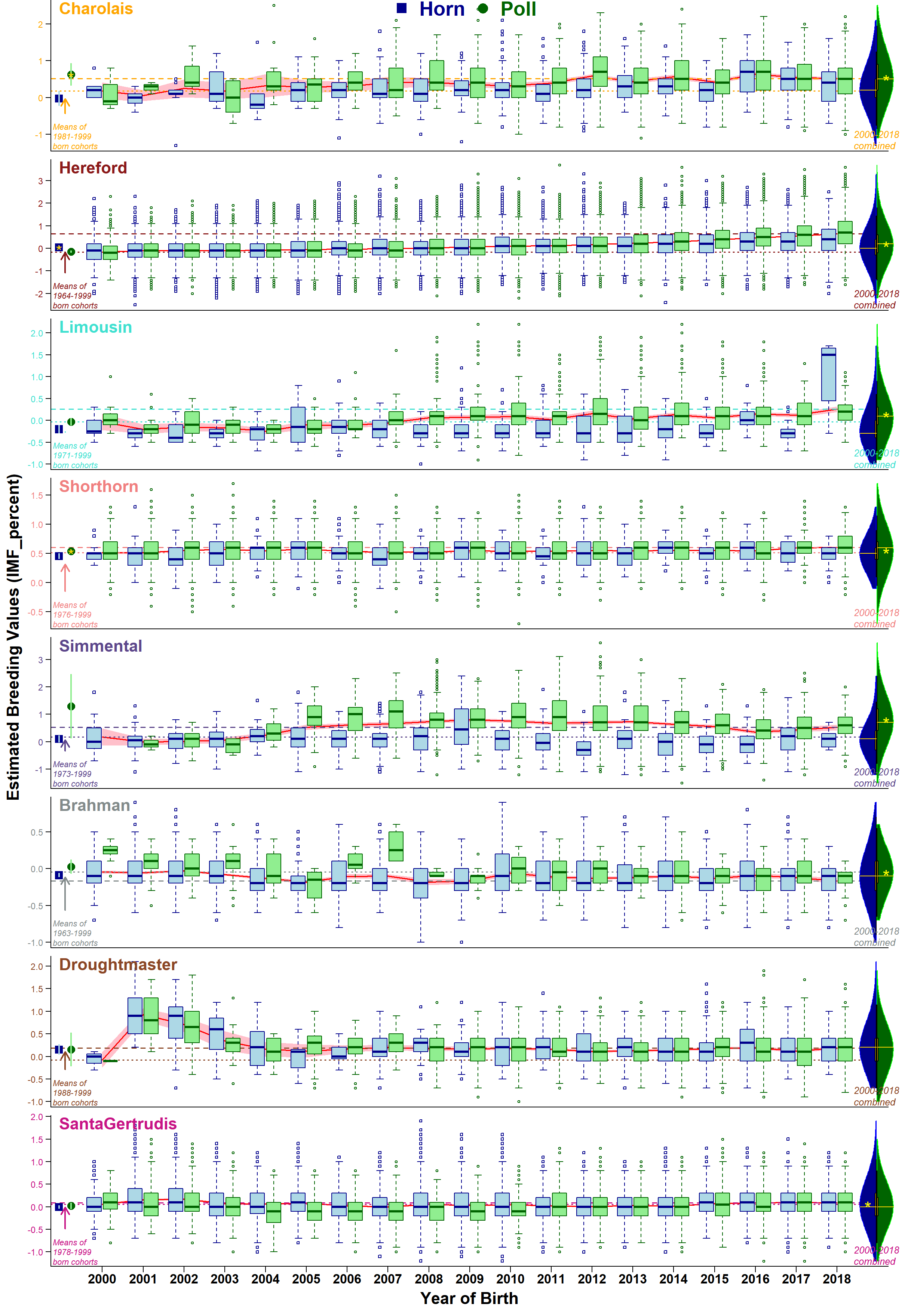


**Figure S8**. Boxplots of intra-muscular-fat EBVs (accuracy ≥ 50%) for horn and poll cohorts born 2000-2018.


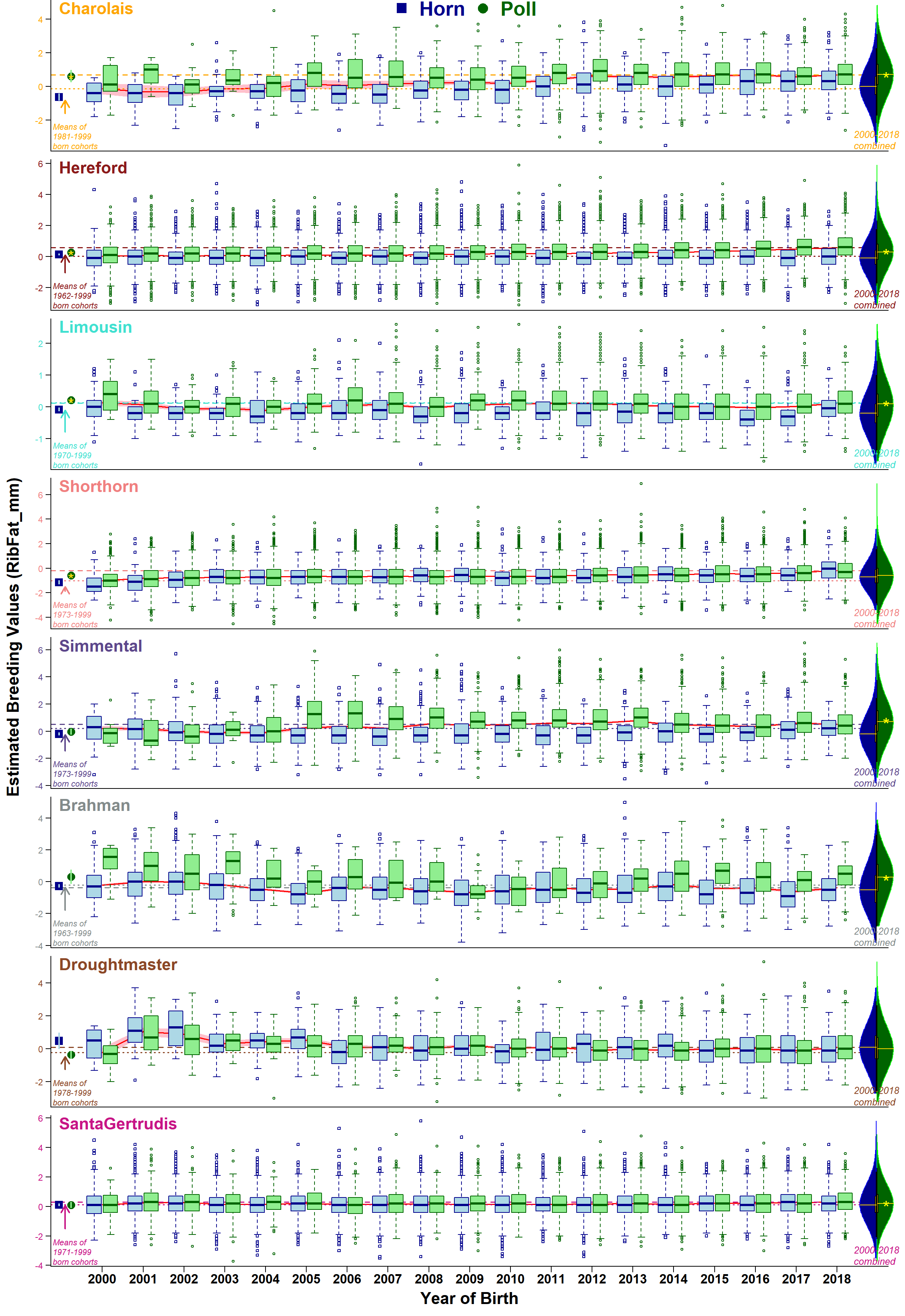


**Figure S9**. Boxplots of rib fat EBVs (accuracy ≥ 50%) for horn and poll cohorts.


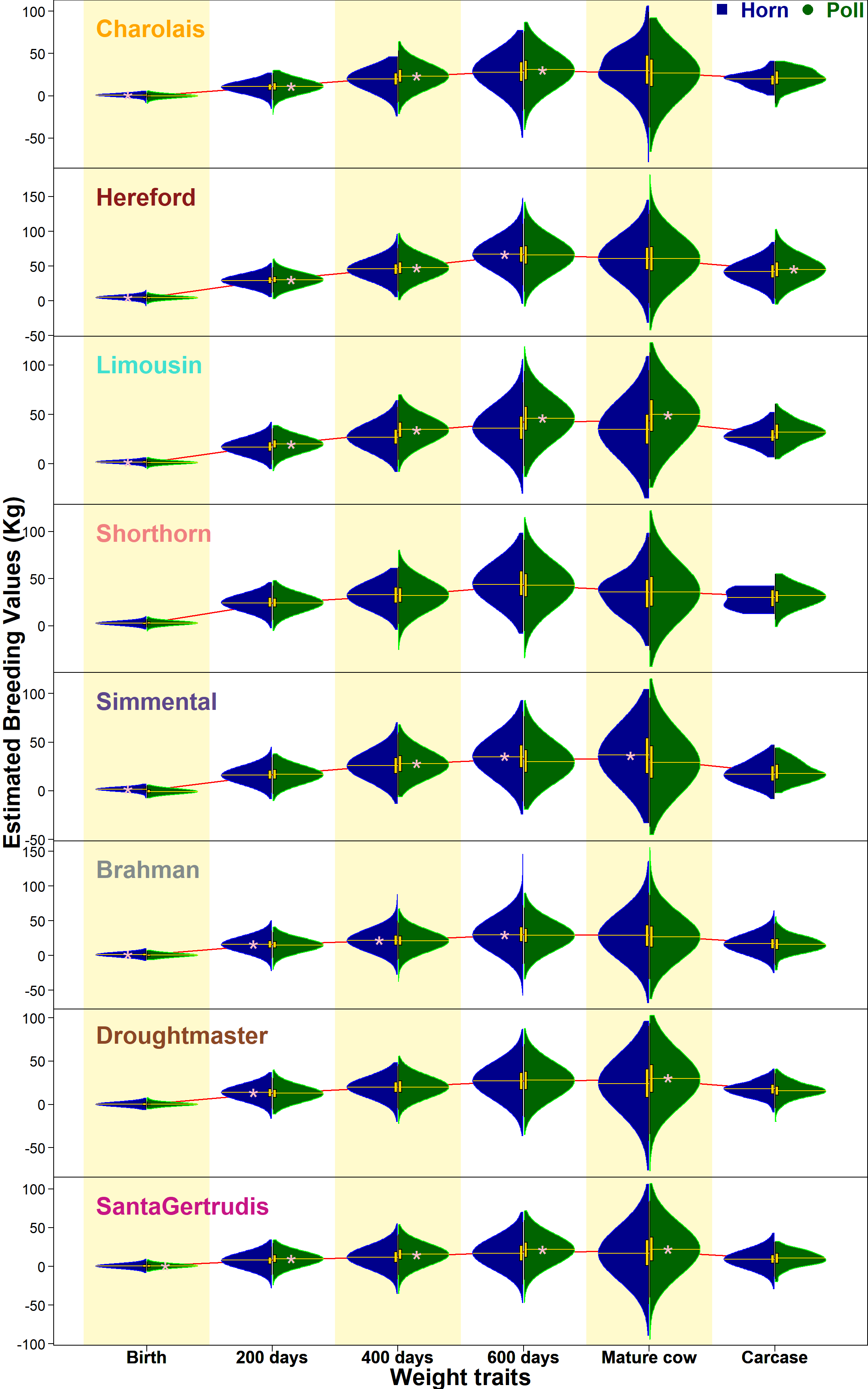


**Figure S10.** Violin-plots of BREEDPLAN EBVs (accuracy ≥ 70%) of weight traits at different life-stages in eight breeds comparing horn and poll cohorts. Pink steric (*) represent a statistically significant higher mean at 70% accuracies of EBVs.


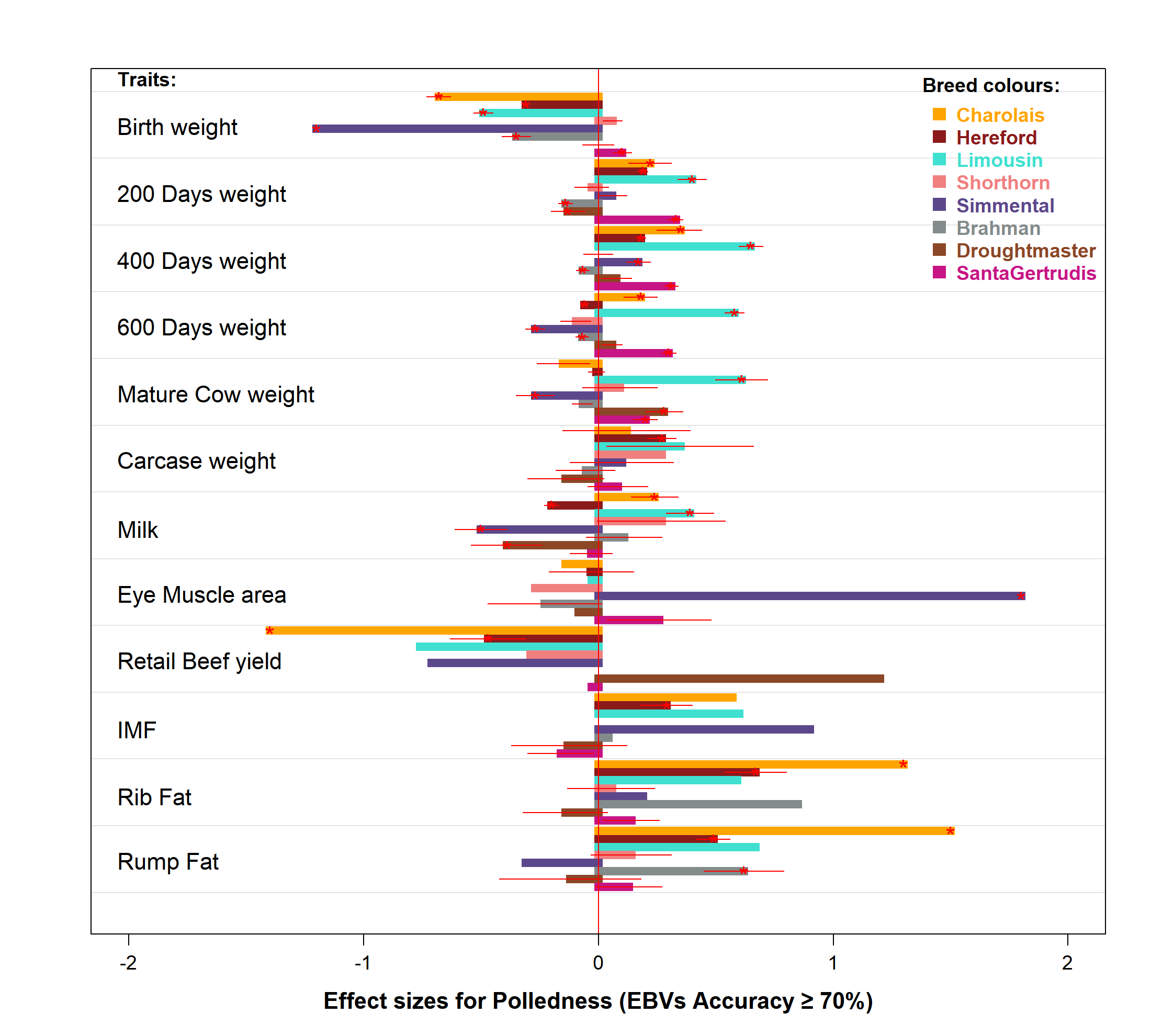


**Figure S11**. Effect sizes (Cohen’s *d*) of polledness in 8 breeds using BREEDPLAN EBVs (accuracy ≥ 70%) on 12 production traits. Red-lines within each bar show 95% confidence intervals of effect sizes. Statistically significant results are shown with a red-star.
